# Recipient bone marrow assimilates the myeloid/lymphoid reconstitution of distinct fetal hematopoietic stem cells

**DOI:** 10.18632/oncotarget.22479

**Published:** 2017-11-15

**Authors:** Xiao-Lin Guo, Lei Chu, Fang Ke, Li-Li Mu, Zhen Li, Jie-Jing Cai, Huai-Fang Li, Deng-Li Hong

**Affiliations:** ^1^ Key Laboratory of Cell Differentiation and Apoptosis of Ministry of Education, Department of Pathophysiology, Shanghai Jiao Tong University School of Medicine (SJTU-SM), Shanghai 200025, China; ^2^ Departments of Gynecology and Obstetrics, Shanghai Tongji Hospital, Tongji University School of Medicine, Shanghai 200065, China

**Keywords:** fetal liver, hematopoietic stem and progenitor cell, transplantation, differentiation, bone marrow

## Abstract

The fetal liver (FL) is a source of hematopoietic stem and progenitor cells (HSPCs) for transplantation. However, whether FL-HSPCs collected at distinct developmental stages reconstitute similarly or differently in the recipient bone marrow (BM) remains undetermined. We examined this problem in a congeneic mouse transplantation model. We first analyzed the lineage components of FL from 12.5 days post-fertilization (dpf) to 18.5 dpf. The myeloid and lymphoid cells were dynamic in absolute number and ratio. The largest difference was between 12.5 and 16.5 dpf. The FL-HSPCs (Lin^−^CD150^+^CD48^−^) at these two time points were then respectively transplanted into the recipients. The difference in lineage reconstitution was undetectable at week 4 or 6 post-transplantation and afterward, indicating that the BM environment assimilated the transplanted cells. Profiling lineage-regulation genes of input and output HSPCs showed that the expression levels were much different in the former and almost the same in the engrafted HSPCs. Therefore, the recipient BM microenvironment could determine the developmental lineage-trends of FL-HSPCs.

## INTRODUCTION

Lack of proper bone marrow (BM) donors remains a major issue in hematopoietic stem cell transplantation therapy, particularly in unexpected disasters, such as nuclear crisis. Alternative sources of hematopoietic stem and progenitor cells (HSPCs) have been proposed, which include the fetal liver (FL) [1, 2]. FL is the primary fetal hematopoietic organ in which HSPCs undergo expansion and differentiation before migrating to the BM at the perinatal stage [3]. FL-HSPCs are dynamic in terms of numbers and differentiation potential for myeloid or lymphoid cells during development. Therefore, given that FL is used as a source of HSPCs for transplantation, a key issue would be whether FL-HSPCs collected at distinct developmental stages reconstitute similarly or differently in the recipient BM. To address this issue, a congeneic mouse transplantation model was used in this study.

To date, HSPC development has been well characterized in detail in mouse, which is used as a model for the human hematopoietic system in many aspects [4]. FL does not produce HSPCs de novo. HSPCs migrate into FL at 11.5 days post-fertilization (dpf) and expand in the organ thereafter. After 12.5 dpf, FL develops into the main fetal hematopoietic organ. The number of HSPCs in FL peaks by 16.5 dpf and maintains a short plateau before starting to decline as they migrate. At different periods, FL is rich in single-lineage HSPCs [4]; in other words, HSPCs at different stages have variant lineage-bias in terms of differentiation [5]. This difference is reflected by the dynamics in lineage distribution of nucleated blood cells, roughly in the ratio of myeloid cells (granulocytes and monocytes, GMs) and lymphoid cells (B and T cells). The difference and dynamics may become an issue when FL is used as a HSPC source for transplantation therapy and research because consistency is required. In this study, we immunophenotypically, functionally, and molecularly characterized the developmental differences in mouse FL-HSPCs and their reconstitution profile in the same recipient BM environment.

## RESULTS

### Dynamics of hematopoietic components of FLs in development

**Figure 1:**
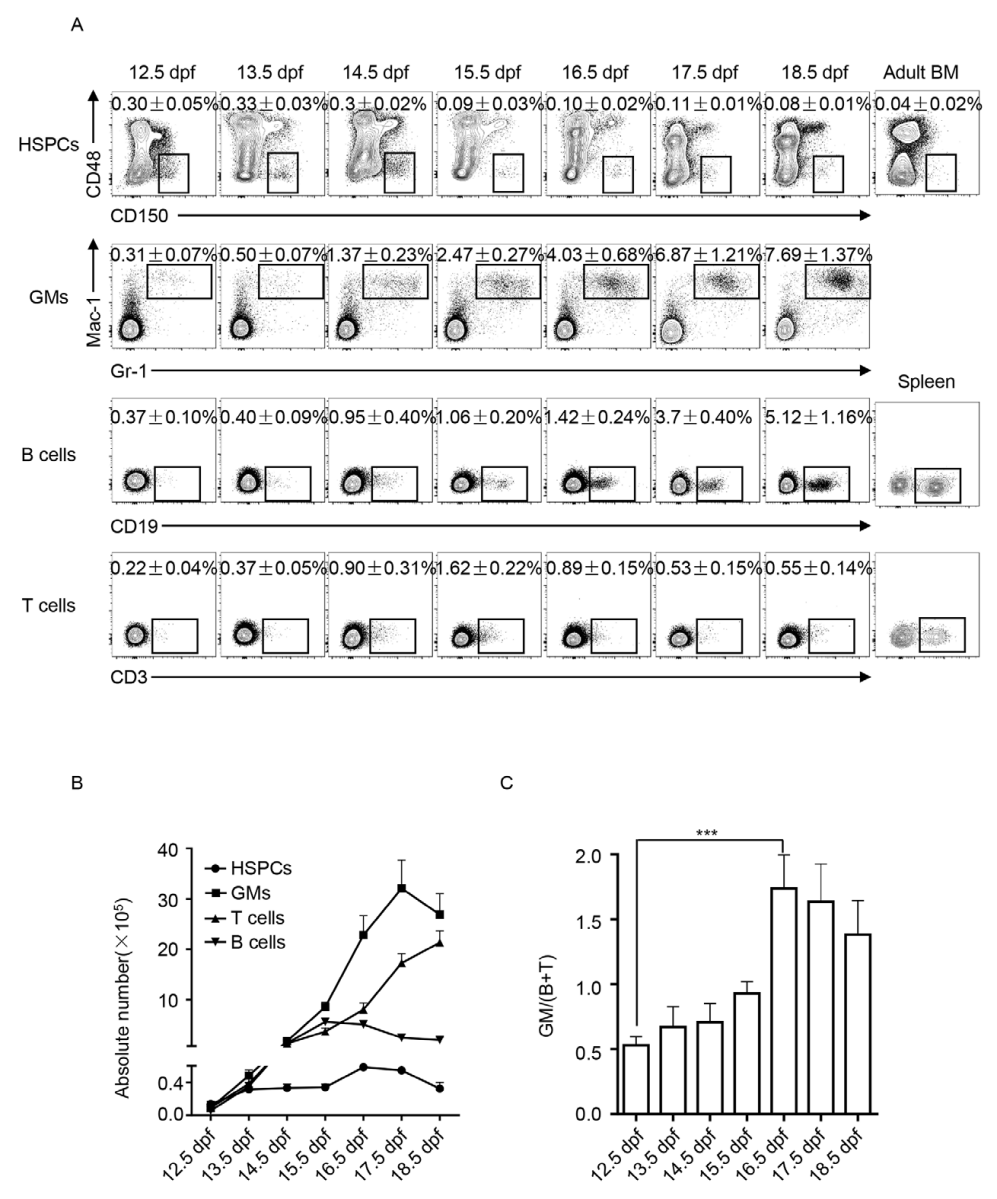
Dynamic analysis of hematopoietic components of fetal liver (FL) from 12.5 dpf to 18.5 dpf **(A)** Flow cytometric analysis of hematopoietic cells in FL: hematopoietic stem and progenitor cells (HSPCs) in the phenotype of Lin^―^CD150^+^CD48^―^, granulocytes and monocytes (GMs) expressing Gr-1 and Mac-1, B cells expressing CD19, and T cells expressing CD3. Data are the mean ± SD of more than three separate experiments. **(B)** Dynamics of absolute numbers of HSPCs, GMs, B, and T cells in FL calculated based on the data in A. **(C)** Ratio between myeloid GM cells and lymphoid B and T cells (GM/(B + T)) was calculated and statistically presented. The difference between 12.5 and 16.5 dpf is the largest, ^***^ *P* < 0.0001.

**Figure 2:**
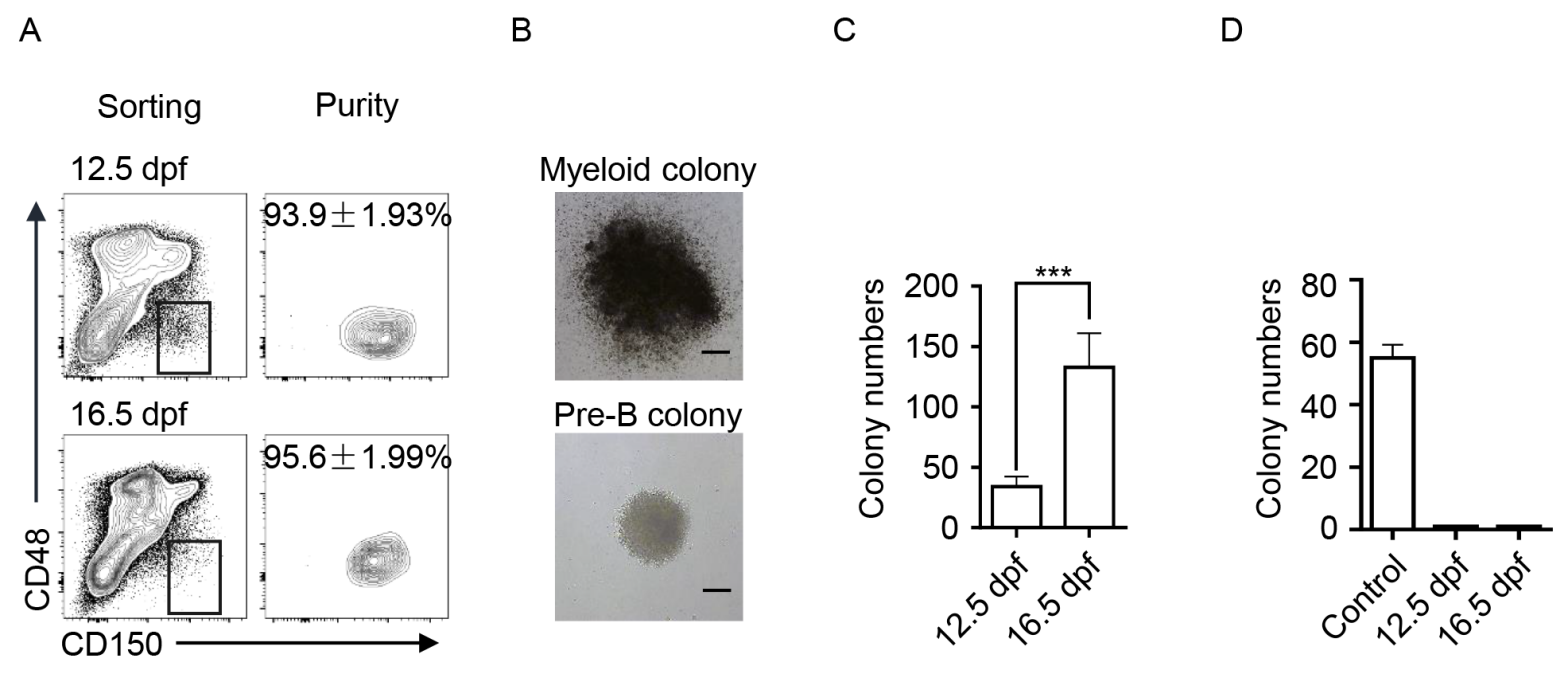
The differentiation potential of FL-HSPCs from 12.5 and 16.5 dpf in CFC assays **(A)** Flow-sorting and purity detection of FL-HSPCs at 12.5 and 16.5 dpf in the phenotypes Lin^−^, CD48^−^, and CD150^+^. **(B)** The typic photographs of a myeloid colony and a pre-B colony. scale bar: 50μm. **(C)** The results of the myloid CFC assay. The colony numbers of 1000 FL-HSPCs plated in the assays were statistically presented, ^***^ *P* < 0.0001. **(D)** The results of Pre-B CFC assay. The colony numbers of 1000 FL-HSPCs and 2×10^5^ adult BM cells (control) were presented.

We comparatively analyzed the hematopoietic components of FLs from 12.5 dpf to 18.5 dpf. Immunophenotypes of HSPCs (Lin^−^CD150^+^CD48^−^), myeloid cells (GMs expressing Gr-1 and Mac-1) and lymphoid cells (B cells expressing CD19 and T cells expressing CD3) were detected via flow cytometry. Percentages of HSPCs, GMs, B, and T cells in total fetal liver cells were dynamically changed at varying trends (Figure 1A). The dynamic changes in the absolute numbers of HSPCs, GMs, B, and T cells in the FLs are shown in Figure 1B. Accordingly, the ratio between myeloid and lymphoid cells, GM/(B + T), was calculated and comparatively shown in Figure 1C. The most significant difference was observed between 12.5 and 16.5 dpf, which indicates that FL-HSPCs at 16.5 dpf might process more potential to develop myloid cells. To functionally prove this assumption, the FL-HSPCs at these two time points were thus collected and purified (Figure 2A) to assess their differentiation potential using CFC assays specific to either myeloid or B-lymphoid progenitor cells. We used the adult bone marrow cells as the technique control of Pre-B CFC assay. The typic photographs of a myeloid colony and a Pre-B colony were shown in Figure 2B. The results showed that the HSPCs from 16.5 dpf FL had increased myeloid differentiation potential compered with the HSPCs of 12.5 dpf FL (Figure 2C). The HSPCs of both time points could not develop any colonies in the lymphoid setting (Figure 2D). Together FL-HSPCs at distinct developmental stages have much varying differentiation potential of myloid or lymphoid cells.

### Recipient BM assimilated the hematopoietic repopulation of distinct FL-HSPCs

**Figure 3:**
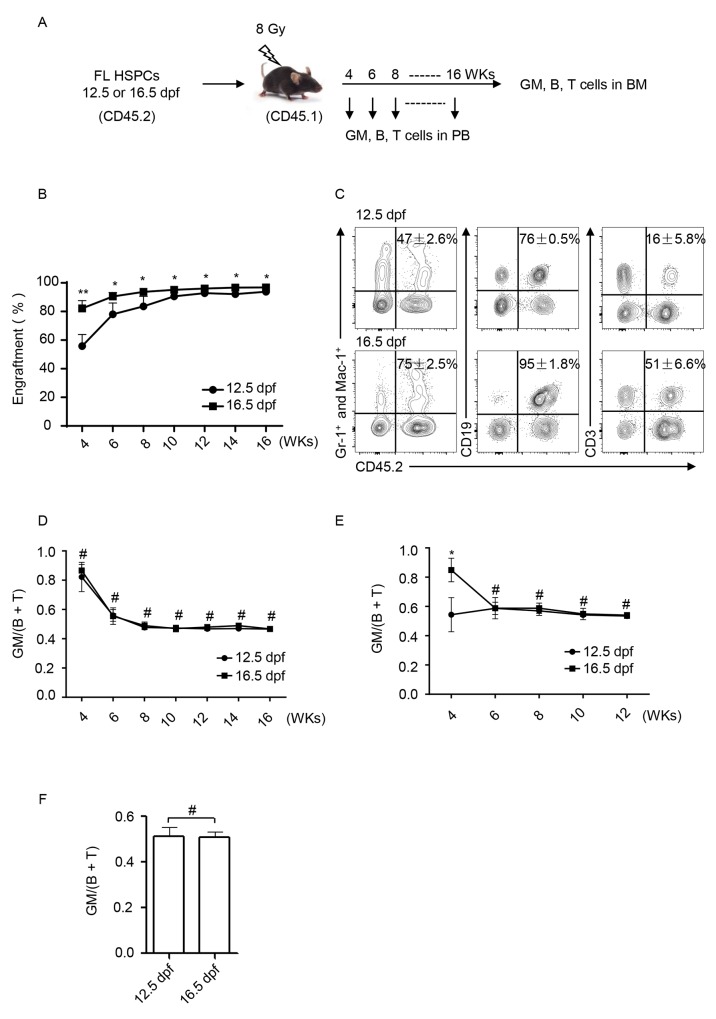
Lineage reconstitution of FL-HSPCs at 12.5 and 16.5 dpf in congeneic recipient mice **(A)** Experimental scheme. PB, peripheral blood; BM, bone marrow; wks, weeks. **(B)** Engraftemts were dynamically analyzed from the PB cells based on the expression of CD45.2 by using flow cytometry,^**^ *P* < 0.005, ^*^ *P* < 0.05. **(C)** Flow cytometric analysis of blood cell reconstitution (CD45.2^+^) of HSPCs at 12.5 and 16.5 dpf FL in recipient PB. A set of representative plots at week 6 post-transplantation was presented. The percents of engrafted cells (CD45.2^+^) in the total GM (Gr-1^+^ and Mac-1^+^), B (CD19^+^), or T (CD3^+^) cells in the recipient PB are shown. **(D)** Dynamic comparison of the GM/(B + T) ratio of the engrafted cells in recipient PB from 5000 HSPCs setting. ^#^ *P* > 0.05. **(E)** Dynamic comparison of the GM/(B + T) ratio of the engrafted cells in recipient PB from 1000 HSPCs setting. ^*^ *P* < 0.05, ^#^ *P* > 0.05. **(F)** At week 16 post-transplantation, the recipient mice were sacrificed, and BM cells were harvested for flow cytometric analysis of lineage reconstitution. The GM/(B + T) ratio was calculated and statistically presented. ^#^ *P* = 0.8123.

The developmental assessments of HSPCs from 12.5 and 16.5 dpf FL in the recipient BM were conducted as outlined in Figure 3A. One thousand or 5000 purified HSPCs (CD45.2^+^) (Figure 2A) were respectively transplanted into recipient mice (CD45.1^+^) that were pre-treated with lethal irradiation (8 Gy). At weeks 3, 4, 6, 8 and 16 post-transplantation, the peripheral blood (PB) was collected, and nucleated blood cells were immunophenotypically analyzed to assess hematopoietic reconstitution. First the engraftment was analyzed based on the expression of CD45.2. When same numbers of HSPCs (5000 cells per mouse) were transplanted to recipients, 16.5 dpf FL-HSPCs reconstituted with higher engraftments than 12.5 dpf FL-HSPCs. But the difference became smaller and smaller with weeks post-transplantation (Figure 3B). In the engrafted WBCs (CD45.2^+^), GMs (Gr-1^+^ Mac-1^+^), B cells (CD19^+^), and T cells (CD3^+^) were then be detected. At week 3, GMs but not B and T cells could be detected. From week 4, both GMs, B and T cells could be detected. A set of representative plots at week 6 is shown (Figure 3C). Accordingly, the ratio between GM and B + T was calculated. Surprisingly, the results in the 5000 HSPCs setting showed no difference at all time points between the HSPCs at 12.5 and 16.5 dpf (Figure 3D). And the results in the 1000 HSPCs setting showed some difference at week 4 but no difference from week 6 (Figure 3E). Consistent with this finding, BM cells of the recipients were harvested and analyzed at 16 weeks post transplantation; hematopoietic lineage distribution appeared the same (Figure 3F). Collectively, the data showed that the BM microenvironment of recipients assimilated the hematopoietic repopulation of FL-HSPCs collected at distinct post-fertilization periods.

### Lineage-regulation gene expression levels in distinct FL-HSPCs were similar in the recipient BM

**Figure 4:**
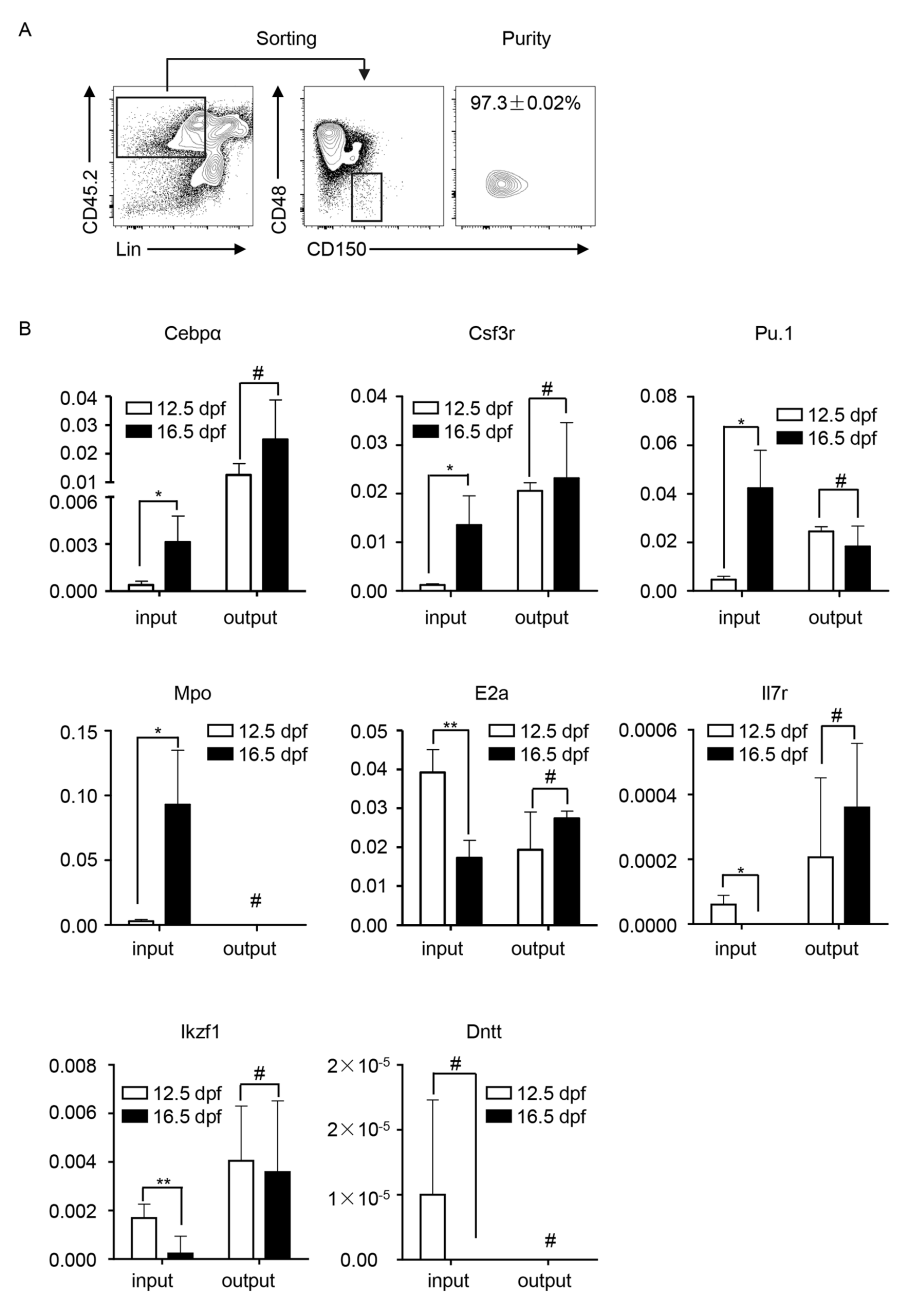
Transcriptional analysis of myeloid and lymphoid cell-regulation genes in the input and output HSPCs in the recipients **(A)** Flow-sorting and purity detection of engrafted HSPCs in the phenotypes Lin^−^, CD48^−^, and CD150^+^ in the recipient BM at week 16 post-transplantation. **(B)** The gene expression was detected by using RT-qPCR. *Cebpα*, *Csf3r*, *PU.1*, and *Mpo* are myeloid cell regulation genes; *E2A, Il7r, Ikzf1, Dntt* are lymphoid cell regulation genes. All data was presented as the mean ± SD. ^*^ *P* < 0.05, ^**^ *P* < 0.005, ^#^ *P* > 0.05.

We then compared the expression of lineage-regulation genes in the input and output HSPCs (Lin^−^CD150^+^CD48^−^) in the recipients to molecularly confirm the assimilation in the recipient BM. The input HSPCs were flow-sorted from the FL at 12.5 dpf and 16.5 dpf respectively (Figure 2A). The output HSPCs were flow-sorted from the engfrated cells (CD45.2^+^) in the recipient (CD45.1^+^) BM at week 16 post-transplantation (Figure 4A). The gene expression was detected by using reverse-transcriptase quantitative polymerase chain reaction (RT-qPCR). The results showed that the expression level of myeloid cell-regulation genes (*Cebpα*, *Csf3r*, *PU.1*, and *Mpo*) in FL-HSPCs at 12.5 dpf was higher than that in FL-HSPCs at 16.5 dpf, and the expression level of lymphoid cell-regulation genes (*E2a, Il7r, Ikzf1* and *Dntt*) indicated the reverse trend. The expression levels of these genes were almost similar in the engrafted HSPCs whether the input cells were obtained from 12.5 or 16.5 dpf FLs (Figure 4B).

Therefore, the BM microenvironment of the recipients determined the lineage trends of differentiation of the donor FL-HSPCs at different stages.

## DISCUSSION

Consistent predictable outcome is important when using HSPCs in stem cell transplantation therapy and research. FL is one of the sources used for such purposes. However, FL-HSPCs are highly dynamic during development in terms of number [3], maturation [6], lineage-trend of differentiation [5], and transcriptional profile [7]. The dynamics might be a potential concern when FL-HSPCs are collected at different developmental stages for transplantation. Our observations in this study partially addressed this issue by showing that FL-HSPCs at different development stages (12.5 dpf vs. 16.5 dpf) reconstituted hematopoiesis similarly in the recipient BM. The BM environment assimilated the differentiation potential for myeloid and lymphoid cells (Figure 3) and the underlying transcription of the related genes (Figure 4). Our data also indicated that the host BM environment could reprogamed the lineage-specfic transcriptions that were primed in the primitive HSPCs [8, 9].

The microenvironment (termed as niche) has been recognized and demonstrated as a key determinant of HSPC fates in hematopoiesis and leukemogenesis [10–13]. Meanwhile, the cellular and molecular compositions of BM niches have been well studied [14–17]; the niches in fetal hematopoietic tissues, such as FL and placenta, remain to be investigated in many aspects in spite of several seminal reports [18, 19]. A better understanding of the fetal hematopoietic niches and identification of their compositional cells and molecules are crucial to address some key issues for the enhanced utilization of fetal HSPCs as a stem cell source for therapy and research. For example, how FL-HSPCs expand without the loss of self-renewal potential should be investigated. Knowing how the biological features of FL-HSPCs change when they migrate into and colonize BM environments is also important. Moreover, the processes of how leukemic lesions occur in FL-HSPCs and are retained in BM HSPCs should be determined. Therefore, our observations in this study provide important information on using FL as a stem cell source in therapy and for research in stem cell biology.

## MATERIALS AND METHODS

### Mice

All mice used in this study were kept under specific pathogen-free conditions in compliance with the National Institutes of Health Guide for the Care and Use of Laboratory Animals with approval (SYXK-2003-0026) from the Scientific Investigation Board of Shanghai Jiao Tong University School of Medicine, Shanghai, China. To obtain the embryos, 8–10-week-old C57BL/6J (B6-Ly5.2) male and female mice were mated at night and then separated the next morning. The time for mice separation was designated at 0.5 dpf.

### Flow cytometric analysis of lineage contribution of the whole blood cells in FL

FLs were isolated from the embryos from 12.5 dpf to 18.5 dpf. Cells were dispersed with IMDM (12440053, Gibco), supplemented with 1% BSA (FA016-50G, Gen-view), and filtered through a 70 μm nylon screen (352350, Falcon) to obtain a single cell suspension. FL cells were counted on a hemocytometer and then incubated with allophycocyanin (APC)-conjugated antibodies: B220 (17-0452, eBioscience), CD3 (17-0032, eBioscience), Gr-1 (17-5931, eBioscience), and Mac-1 (17-0112, eBioscience) at 4°C for 20 min. The cells were then washed with PBS and detected on a Fortessa flow cytometer (BD). The flow cytometry data were analyzed using FlowJo 7.6 software.

### Isolation of FL-HSPCs

Progenitor cells were enriched by depleting lineage-positive cells using magnetic cell sorting. Whole FL cells were incubated with biotin-labeled monoclonal antibodies: B220, CD3, Gr-1, and Ter119, all these antibodies are from mouse hematopoietic lineage biotin panel (88-7774-75, ebioscience). Cells were then washed with MACS buffer (PBS, 2 mM EDTA, and 0.5% BSA), followed by staining with streptavidin-conjugated magnetic beads (558451, BD). After staining for 20 min at 4°C, the cells were washed and suspended in MACS buffer, and the lineage-positive cells were depleted by the magnetic system. The lineage-depleted progenitor cells were then incubated with APC-labeled lineage markers, including B220 (17-0452, eBioscience), CD3 (17-0032, eBioscience), Gr-1 (17-5931, eBioscience), Mac-1 (17-0112, eBioscience), PE-labeled anti-CD150 antibody (12-1502, eBioscience), and FITC-labeled anti-CD48 antibody (11-0481, eBioscience). After incubation for 30 min at 4°C, the cells were washed with PBS, suspended with IMDM, and supplemented with 1% BSA. Flow-sorting was performed on an LSR Fortessa flow cytometer (BD).

### Transplantation assay

Adult recipient mice (10-week-old C57BL/Ka-CD45.1:Thy-1.2 mice) were irradiated with a gamma ray source delivering approximately 3 Gy/min at a dose of 8 Gy. HSPCs (Lin^−^CD150^+^CD48^−^) from a relevant donor (CD45.2^+^) were sorted as mentioned earlier and then injected into the tibia of lethally irradiated CD45.1^+^ recipients. The number of injected cells per mouse was 5,000. Peripheral blood samples were obtained from the tail veins of the recipient mice starting at the fourth week after transplantation. The blood was subjected to ammonium chloride potassium red-cell lysis buffer (NH4CL 3.735 g; Tris 1.3 g per 500 mL), and the cells were washed with PBS after 10 min at room temperature. To monitor donor cell engraftment and lineage distribution, the cells were stained with PE-labeled anti-CD45.2 (12-0454, eBioscience), APC-labeled anti-B220 (17-0452, eBioscience), APC-labeled anti-CD3 (17-0032, eBioscience), APC-labeled anti-Mac-1 (17-0112, eBioscience), and APC-labeled anti-Gr-1 (17-5931, eBioscience). The contributions of the injected (donor) cells to the populations of circulating GM, B, and T cells were calculated as previously described [20].

### Gene expression analysis by reverse-transcriptase quantitative polymerase chain reaction (RT-qPCR)

Total RNA was extracted from 1×10^4^ to 1×10^5^ highly purified HSPCs by Tri Pure Isolation Reagent (11667165001, Roche), as recommended by the manufacturer. The extracted RNA was treated with RQ1 RNase-free DNase (M6101, Promega). Complementary DNA was generated by M-MLV reverse transcriptase (M1705, Promega). RT-qPCR was carried out using SYBR Green PCR Master Mix (4309155, Applied Biosystems). Threshold cycle (Ct) was assessed by determining the cycle number using the sequence detector system ABI PRISM 7900 (Applied Biosystems). All gene expression results were normalized to the expression of the housekeeping gene *β-actin*. The data were presented as the mean ± SD of three samples. Primer sequences were as follows: *Dntt*: forward primer 5’-CATGTGCCCCTATGATCGCC-3’ and reverse primer 5’-AGAAACACCCTCTTAGTCCTGT-3’; *E2a*: forward primer 5’-CATAACCATGCCAGCCTCCC-3’ and reverse primer 5’-TCCTCTTTCTCCTCCCGCTT-3’; *Il7r*: forward primer 5’- GCGGACGATCACTCCTTCTG-3’ and reverse primer 5’- AGCCCCACATATTTGAAATTCCA-3’; *Ikzf1*: forward primer 5’- CTGTGTGATGAAGTGCGCTG-3’ and reverse primer 5’- CAGGAAGCTGGGGTAACAGG-3’; *Csf3r*: forward primer 5’-GGGTCCACCAACAGTACAGG-3’ and reverse primer 5’-GAGCCAGGTCACTACACAGG-3’; *Mpo*: forward primer 5’-GCTTCCAAGACAATGGCAGGG-3’ and reverse primer 5’-GGTGTGCATGGAGGTGAGC-3’; *PU.1*: forward primer 5’-CTCACCGCCCCTCCATC-3’ and reverse primer 5’-GTGTGCGGAGAAATCCCAGTA-3’; *Cebpα*: forward primer 5’-GCAAAGCCAAGAAGTCGGTG-3’ and reverse primer 5’-TCACTGGTCAACTCCAGCAC-3’; and *β-actin*: forward primer 5’-GGCTCCTAGCACCATGAAGA-3’ and reverse primer 5’-GGGTGTAAAACGCAGCTCAG-3’.

### CFC assay

Assays were performed in MethoCult™ GF M3434 and M3630 medium as the protocol described. Cells were plated in 35 mm dishes containing methylcellulose. The colonies were scored under a microscope 13–14 days post plating.

### Statistics

All data are represented as the mean ± SD. Comparisons between two samples were conducted using the unpaired Student’s *t*-test. Statistical significance was considered at *P* < 0.05, and all statistical analyses were performed with GraphPad Prism 6.
